# Supplementary material for: Derived Neutrophil-Lymphocyte Ratio and C-Reactive Protein as Prognostic Factors for Early-Stage Non-Small Cell Lung Cancer Treated with Stereotactic Body Radiation Therapy
Source: Diagnostics (Basel). 2023 Jan 14;13(2):313. doi: 10.3390/diagnostics13020313 (PMC9857614; doi:10.3390/diagnostics13020313)
Supplement: Supplementary file 1 [file diagnostics-13-00313-s001.zip › diagnostics-2114750-supplementary/Supplement Figure Legends.pdf]

Supplement Figure S1 Pre-treatment distribution of white blood cells (WBC), neutrophils, lymphocytes, monocytes, platelets, albumin, LDH, and CRP.

Supplement Figure S2 Receiver operating characteristic curves for OS of dNLR, MLR, PLR, albumin, LDH, and CRP.

Supplement Figure S3 Kaplan–Meier curves for LRR and PFS, stratified by CRP group.

Supplement Figure S4 Boxplot of pre- and post-SBRT WBC, neutrophils, lymphocytes, dNLR, albumin, LDH, and CRP.

Supplement Figure S5 Kaplan–Meier curves for cancer-specific survival, stratified by baseline dNLR and CRP group, respectively.

Supplement Figure S6 Kaplan–Meier curves for OS, stratified by Group 1, 2 and 3, respectively.
