# Supplementary material for: Derived Neutrophil-Lymphocyte Ratio and C-Reactive Protein as Prognostic Factors for Early-Stage Non-Small Cell Lung Cancer Treated with Stereotactic Body Radiation Therapy
Source: Diagnostics (Basel). 2023 Jan 14;13(2):313. doi: 10.3390/diagnostics13020313 (PMC9857614; doi:10.3390/diagnostics13020313)
Supplement: Supplementary file 1 [file diagnostics-13-00313-s001.zip › diagnostics-2114750-supplementary/Supplement Tables.pdf]

Supplement Table S1. Cox proportional hazards regression for LRR.

Covariables	Univariable Analysis		Multivariable Analysis	
	**Hazard Ratio (95% CI)**	***P* value**	**Hazard Ratio (95% CI)**	***P* value**
**Age (year)**	1.01 (0.97–1.06)	0.623	1.02 (0.97–1.07)	0.506
**Gender**				
Female	1 [Reference]		1 [Reference]	
Male	2.60 (0.89–7.59)	0.080	2.01 (0.43–9.42)	0.374
**Smoking Status**				
Never smoker	1 [Reference]		1 [Reference]	
Former/current smoker	1.96 (0.82–4.71)	0.130	1.27 (0.37–4.34)	0.699
**T-stage**				
T1	1 [Reference]		1 [Reference]	
T2	0.77 (0.35–1.71)	0.518	0.49 (0.21–1.14)	0.099
**Histologic subtype**				
Adenocarcinoma	1 [Reference]		1 [Reference]	
Squamous cell carcinoma	1.88 (0.68–5.20)	0.223	0.66 (0.20–2.25)	0.509
NSCLC, NOS	0.81 (0.21–3.09)	0.762	0.28 (0.06–1.19)	0.083
No pathologic diagnosis	0.82 (0.30–2.25)	0.696	0.55 (0.18–1.72)	0.304
**BED (Gy)**	0.96 (0.90–1.02)	0.221	0.91 (0.85–0.98)	**0.011**
**dNLR**				
<1.4	1 [Reference]			
≥1.4	1.12 (0.48–2.60)	0.791		
**MLR**				
<0.3	1 [Reference]		1 [Reference]	0.171
≥0.3	2.36 (0.94–5.91)	0.067	2.00 (0.74–5.37)	
**PLR**				
<128	1 [Reference]			
≥128	0.73 (0.33–1.61)	0.435		
**Serum albumin level (g/L)**				
<43.0	1 [Reference]			
≥43.0	0.57 (0.25–1.33)	0.195		
**LDH (U/L)**				
<240	1 [Reference]			
≥240	0.04 (0.00–21.82)	0.323		
**CRP**				
<2.9	1 [Reference]		1 [Reference]	
≥2.9	10.87 (3.73–31.72)	**<0.001**	17.17 (5.56–53.08)	**<0.001**

Supplement Table S2. Cox proportional hazards regression for DM.

Covariables	Univariable Analysis		Multivariable Analysis	
	**Hazard Ratio (95% CI)**	***P* value**	**Hazard Ratio (95% CI)**	***P* value**
**Age (year)**	1.01 (0.97–1.05)	0.639	1.01 (0.97–1.05)	0.605
**Gender**				
Female	1 [Reference]		1 [Reference]	
Male	1.98 (0.91–4.29)	0.084	1.76 (0.64–4.79)	0.271
**Smoking Status**				
Never smoker	1 [Reference]		1 [Reference]	
Former/current smoker	1.65 (0.85–3.19)	0.137	0.90 (0.37–2.16)	0.810
**T-stage**				
T1	1 [Reference]		1 [Reference]	
T2	0.85 (0.46–1.58)	0.604	0.68 (0.35–1.30)	0.243
**Histologic subtype**				
Adenocarcinoma	1 [Reference]		1 [Reference]	
Squamous cell carcinoma	2.25 (1.03–4.95)	**0.043**	1.50 (0.63–3.55)	0.362
NSCLC, NOS	0.90 (0.32–2.57)	0.847	0.55 (0.18–1.65)	0.285
No pathologic diagnosis	0.85 (0.38–1.93)	0.700	0.81 (0.35–1.88)	0.616
**BED (Gy)**	0.91 (0.86–0.97)	**0.004**	0.90 (0.84–0.97)	**0.003**
**dNLR**				
<1.4	1 [Reference]		1 [Reference]	
≥1.4	2.00 (0.95–4.23)	0.068	2.05 (0.95–4.45)	0.069
**MLR**				
<0.3	1 [Reference]			
≥0.3	1.13 (0.60–2.12)	0.705		
**PLR**				
<128	1 [Reference]			
≥128	0.76 (0.41–1.40)	0.375		
**Serum albumin level (g/L)**				
<43.0	1 [Reference]			
≥43.0	0.67 (0.35–1.28)	0.224		
**LDH (U/L)**				
<240	1 [Reference]			
≥240	0.52 (0.13–2.17)	0.371		
**CRP**				
<2.9	1 [Reference]		1 [Reference]	
≥2.9	1.80 (0.98–3.33)	0.191	1.97 (1.04–3.72)	**0.038**

Supplement Table S3. Cox proportional hazards regression for PFS.

Covariables	Univariable Analysis		Multivariable Analysis	
	**Hazard Ratio (95% CI)**	***P* value**	**Hazard Ratio (95% CI)**	***P* value**
**Age (year)**	1.01 (0.98–1.05)	0.464	1.01 (0.97–1.06)	0.597
**Gender**				
Female	1 [Reference]		1 [Reference]	
Male	2.23 (1.08–4.60)	**0.030**	1.74 (0.64–4.75)	0.282
**ECOG PS**				
0	1 [Reference]		1 [Reference]	
1	1.40 (0.69–2.85)	0.348	1.52 (0.70–3.32)	0.297
2–3	0.74 (0.27–2.05)	0.566	0.87 (0.26–2.84)	0.812
**Charlson Comorbidity Index**				
2	1 [Reference]		1 [Reference]	
3–4	1.16 (0.65–2.07)	0.627	0.83 (0.45–1.55)	0.563
≥5	1.29 (0.44–3.77)	0.645	0.52 (0.16–1.72)	0.285
**Smoking Status**				
Never smoker	1 [Reference]		1 [Reference]	
Former/current smoker	1.99 (1.07–3.69)	**0.030**	1.13 (0.48–2.67)	0.785
**Pulmonary Function**				
Normal	1 [Reference]		1 [Reference]	
Mild	0.70 (0.20–2.50)	0.584	0.61 (0.15–2.48)	0.492
Moderate	1.67 (0.60–4.59)	0.324	0.97 (0.31–2.97)	0.952
Severe	1.47 (0.57–3.76)	0.422	0.90 (0.32–2.55)	0.846
Unknown	1.63 (0.63–4.26)	0.315	1.39 (0.47–4.06)	0.550
**T-stage**				
T1	1 [Reference]		1 [Reference]	
T2	0.78 (0.44–1.36)	0.379	0.65 (0.35–1.21)	0.175
**Histologic subtype**				
Adenocarcinoma	1 [Reference]		1 [Reference]	
Squamous cell carcinoma	2.42 (1.15–5.09)	**0.020**	1.28 (0.53–3.08)	0.590
NSCLC, NOS	1.16 (0.46–2.92)	0.749	0.63 (0.23–1.74)	0.369
No pathologic diagnosis	1.08 (0.51–2.26)	0.849	0.89 (0.39–2.03)	0.780
**BED (Gy)**	0.92 (0.87–0.97)	**0.002**	0.89 (0.84–0.94)	**<0.001**
**dNLR**				
<1.4	1 [Reference]		1 [Reference]	
≥1.4	1.77 (0.92–3.41)	0.086	1.85 (0.89–3.87)	0.100
**MLR**				
<0.3	1 [Reference]			
≥0.3	1.32 (0.74–2.35)	0.345		
**PLR**				
<128	1 [Reference]			
≥128	0.82 (0.47–1.43)	0.487		
**Serum albumin level (g/L)**				
<43.0	1 [Reference]		1 [Reference]	
≥43.0	0.60 (0.33–1.10)	0.097	0.86 (0.44–1.66)	0.644
**LDH (U/L)**				
<240	1 [Reference]			
≥240	0.42 (0.10–1.74)	0.233		
**CRP**				
<2.9	1 [Reference]		1 [Reference]	
≥2.9	1.51 (0.82–2.79)	0.191	3.13 (1.63–6.01)	**0.001**

Supplement Table S4. Cox proportional hazards regression for cancer-specific survival.

Covariables	Univariable Analysis		Multivariable Analysis	
	**Hazard Ratio (95% CI)**	***P* value**	**Hazard Ratio (95% CI)**	***P* value**
**Age (year)**	1.03 (0.98–1.07)	0.288	1.01 (0.95–1.07)	0.864
**Gender**				
Female	1 [Reference]		1 [Reference]	
Male	4.20 (1.27–13.87)	**0.019**	2.67 (0.55–13.04)	0.224
**ECOG PS**				
0	1 [Reference]		1 [Reference]	
1	2.20 (0.75–6.41)	0.149	2.76 (0.85–9.03)	0.093
2–3	1.42 (0.36–5.70)	0.618	1.84 (0.35–9.69)	0.474
**Charlson Comorbidity Index**				
2	1 [Reference]		1 [Reference]	
3–4	1.39 (0.63–3.07)	0.410	1.12 (0.47–2.67)	0.796
≥5	1.91 (0.53–6.95)	0.325	0.98 (0.23–4.15)	0.978
**Smoking Status**				
Never smoker	1 [Reference]		1 [Reference]	
Former/current smoker	2.86 (1.17–7.04)	**0.022**	1.12 (0.34–3.64)	0.854
**Pulmonary Function**				
Normal	1 [Reference]		1 [Reference]	
Mild	0.38 (0.04–3.70)	0.408	0.28 (0.02–3.18)	0.301
Moderate	2.48 (0.64–9.62)	0.189	0.71 (0.15–3.35)	0.669
Severe	1.86 (0.51–6.78)	0.344	0.73 (0.17–3.21)	0.682
Unknown	1.76 (0.47–6.62)	0.406	1.29 (0.30–5.53)	0.729
**T-stage**				
T1	1 [Reference]			
T2	1.46 (0.70–3.03)	0.313	1.31 (0.58–2.96)	0.511
**Histologic subtype**				
Adenocarcinoma	1 [Reference]			
Squamous cell carcinoma	2.69 (1.00–7.24)	**0.050**	1.13 (0.37–3.48)	0.828
NSCLC, NOS	1.41 (0.41–4.83)	0.580	1.06 (0.26–4.27)	0.934
No pathologic diagnosis	1.27 (0.47–3.41)	0.639	1.22 (0.41–3.64)	0.723
**BED (Gy)**	0.92 (0.85–0.99)	**0.020**	0.88 (0.81–0.96)	**0.004**
**dNLR**				
<1.4	1 [Reference]			
≥1.4	3.23 (1.12–9.29)	**0.030**	4.02 (1.25–12.96)	**0.020**
**MLR**				
<0.3	1 [Reference]			
≥0.3	1.39 (0.65–3.00)	0.397		
**PLR**				
<128	1 [Reference]			
≥128	0.88 (0.42–1.82)	0.722		
**Serum albumin level (g/L)**				
<43.0	1 [Reference]			
≥43.0	0.48 (0.21–1.07)	**0.073**	0.66 (0.25–1.73)	0.394
**LDH (U/L)**				
<240	1 [Reference]			
≥240	0.87 (0.21–3.65)	0.846		
**CRP**				
<2.9	1 [Reference]			
≥2.9	2.30 (1.10–4.77)	**0.026**	2.87 (1.21–6.80)	**0.017**
