# Supplementary figures and images for: Derived Neutrophil-Lymphocyte Ratio and C-Reactive Protein as Prognostic Factors for Early-Stage Non-Small Cell Lung Cancer Treated with Stereotactic Body Radiation Therapy
Source: Diagnostics (Basel). 2023 Jan 14;13(2):313. doi: 10.3390/diagnostics13020313 (PMC9857614; doi:10.3390/diagnostics13020313)

A

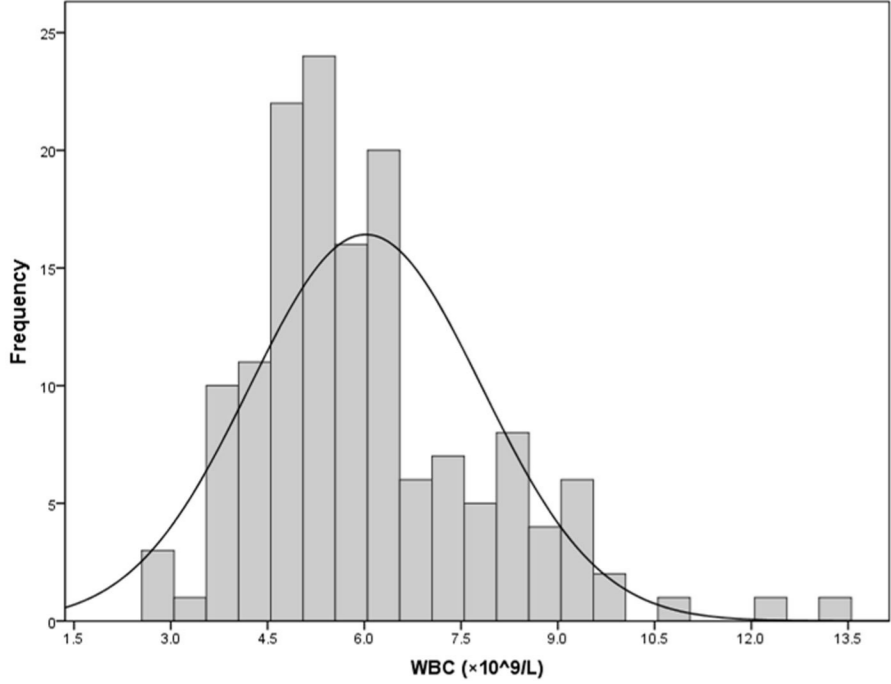

B

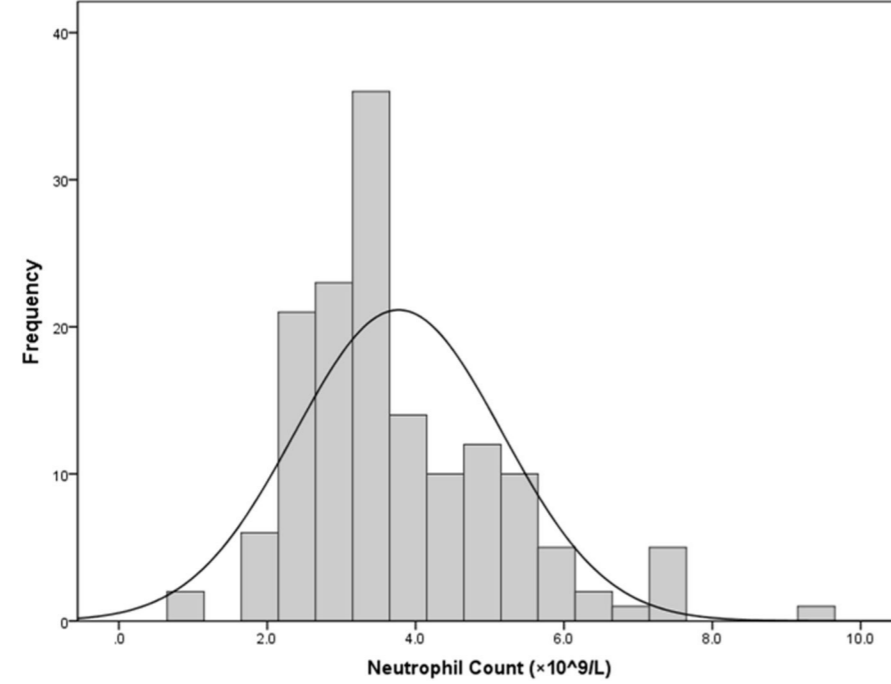

C

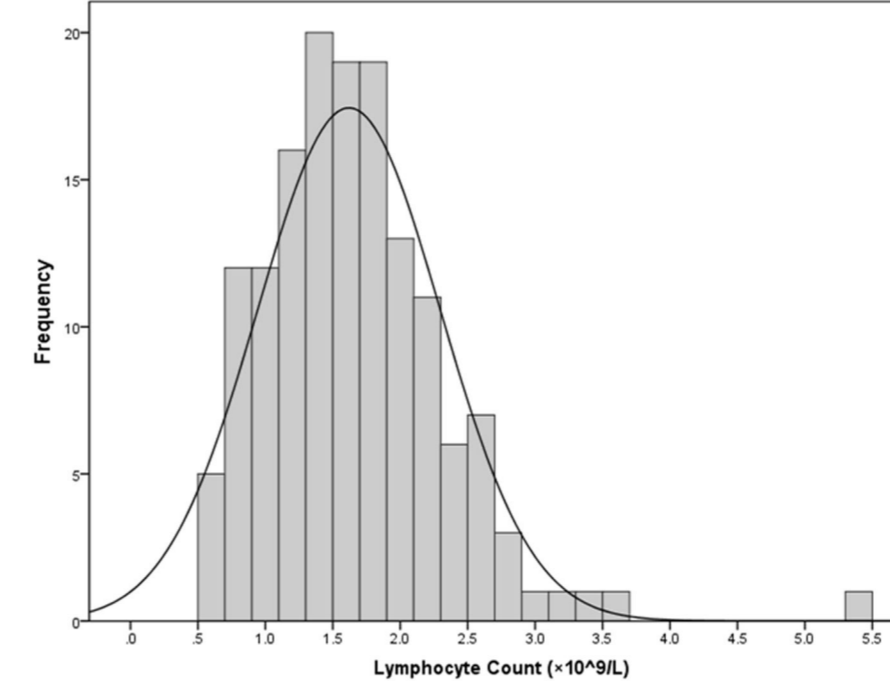

D

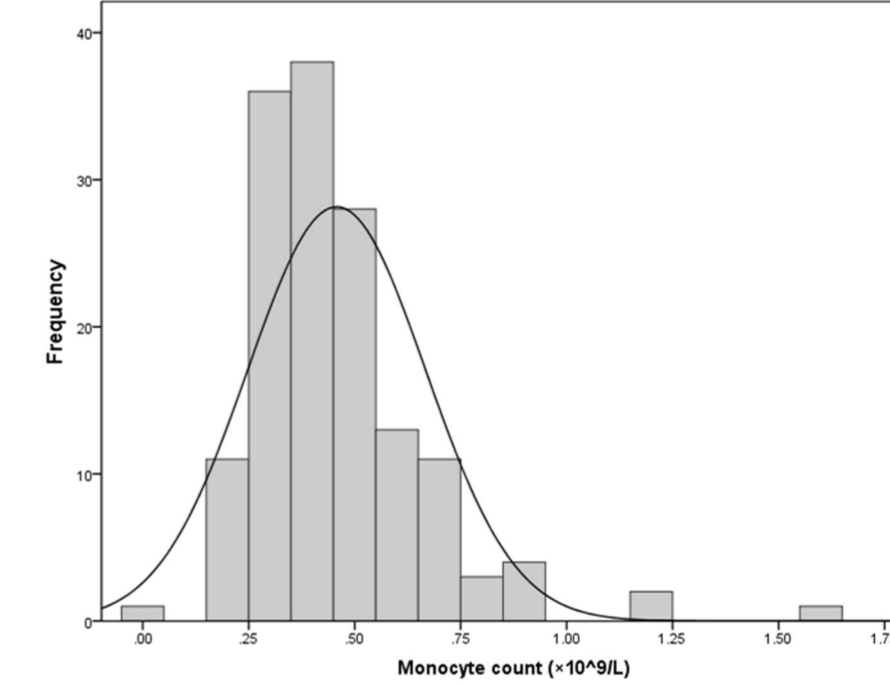

E

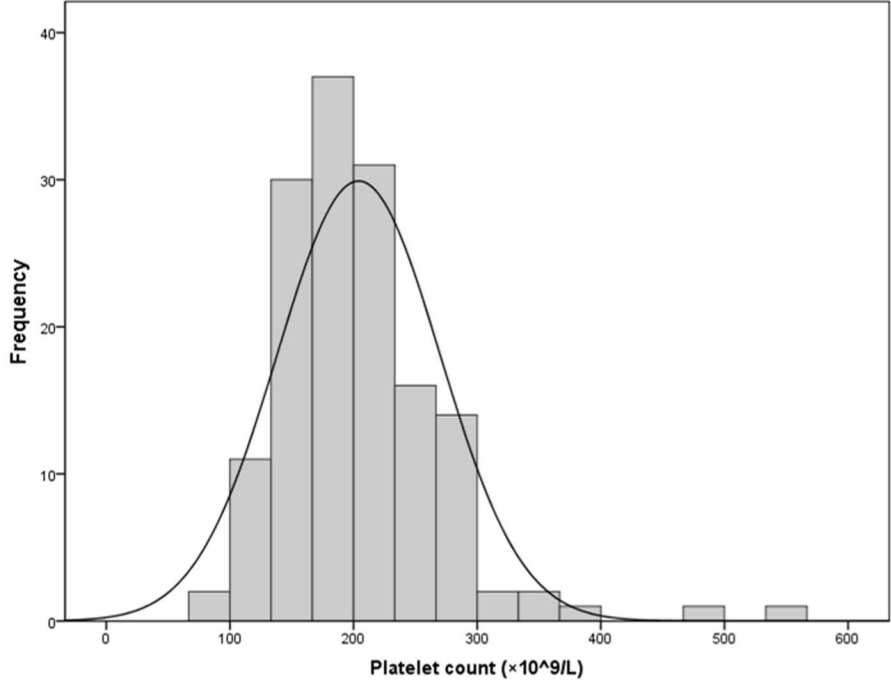

F

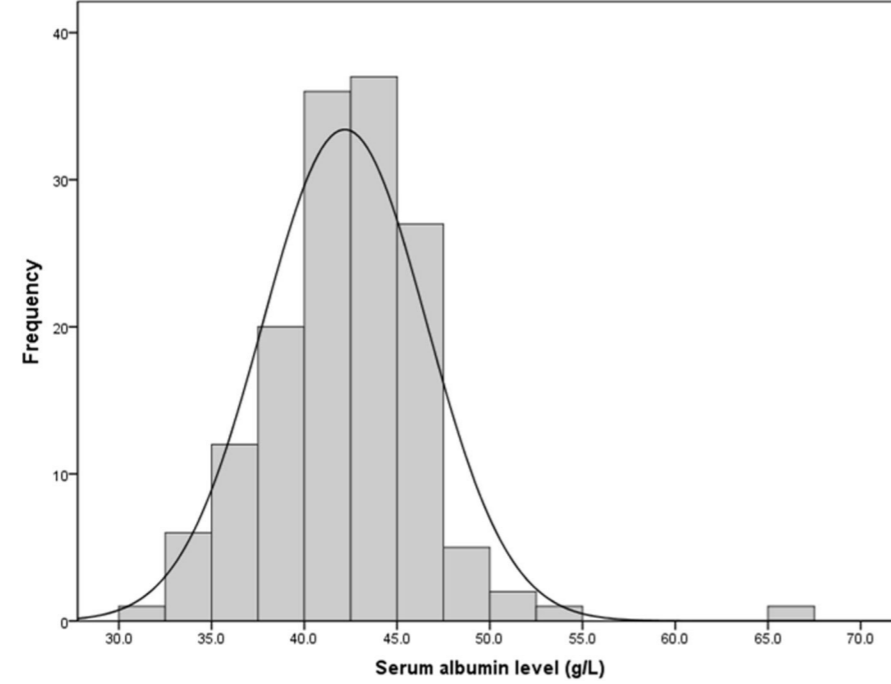

G

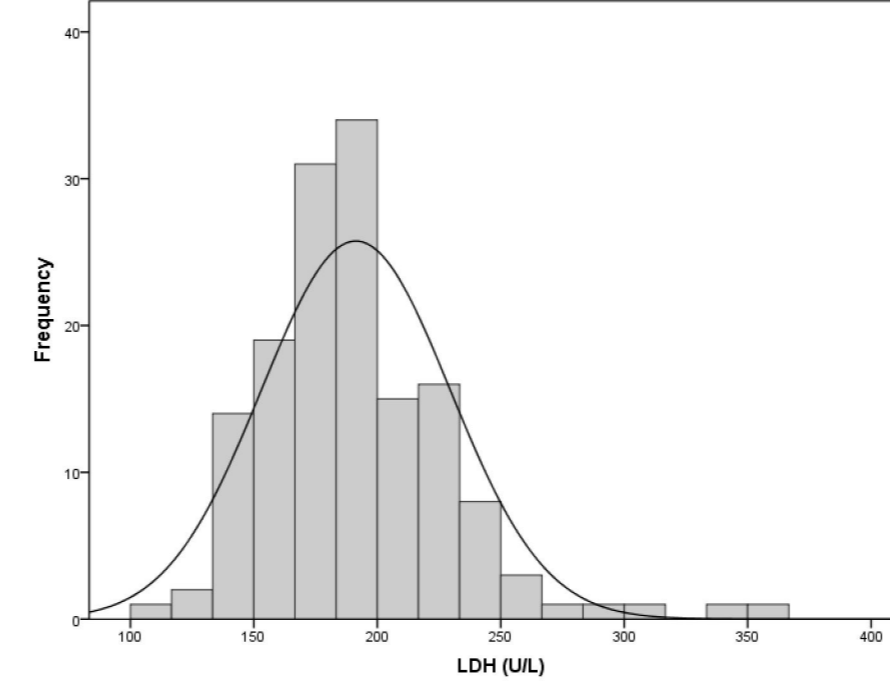

H

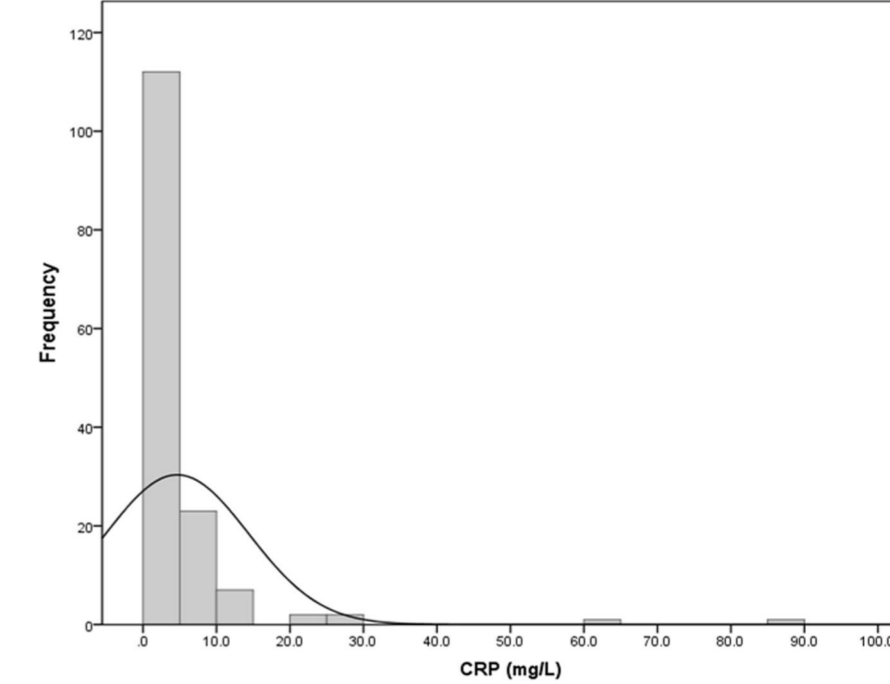

Supplement: Supplementary file 1 [file diagnostics-13-00313-s001.zip › diagnostics-2114750-supplementary/Supplement Figure S1.pdf]

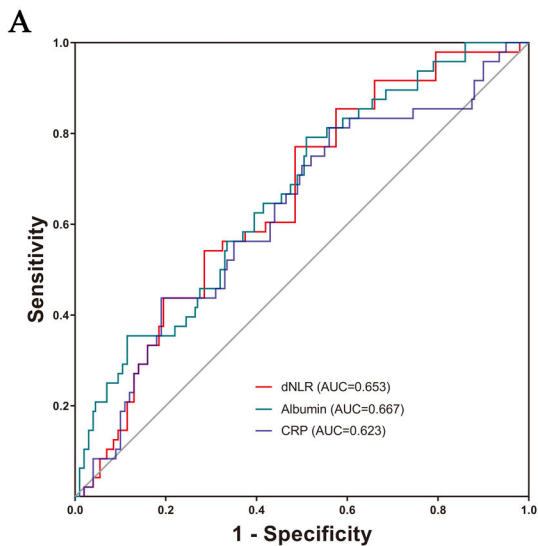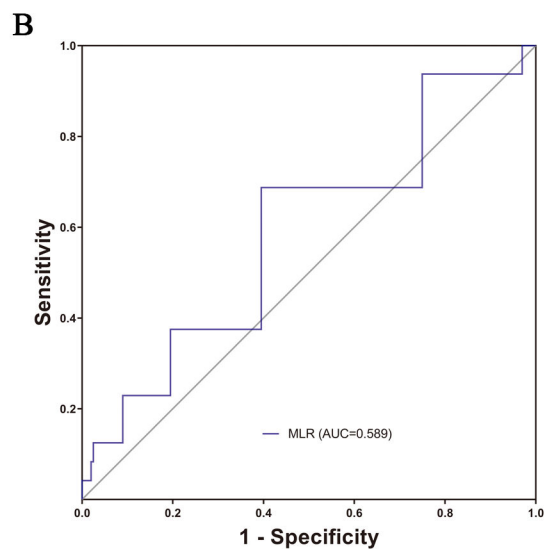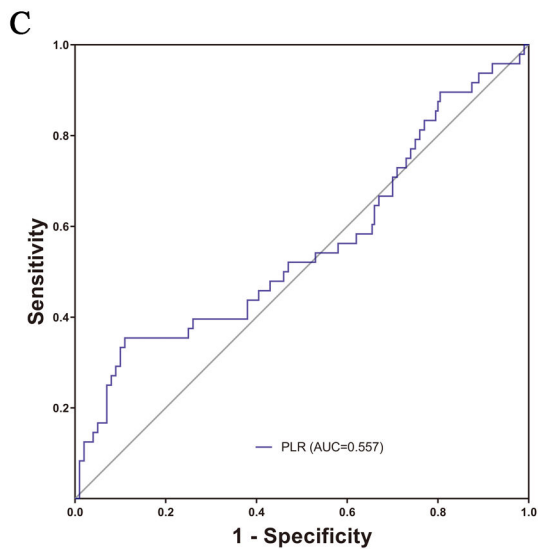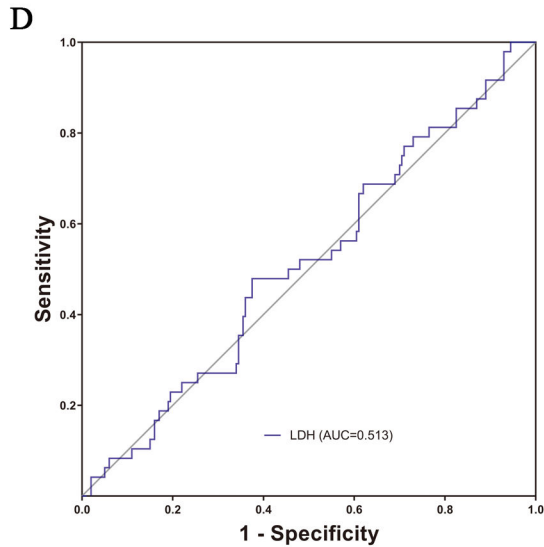

Supplement: Supplementary file 1 [file diagnostics-13-00313-s001.zip › diagnostics-2114750-supplementary/Supplement Figure S2.pdf]

A

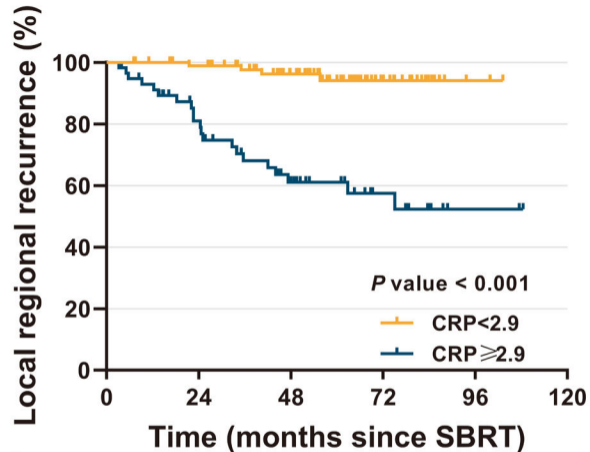

B

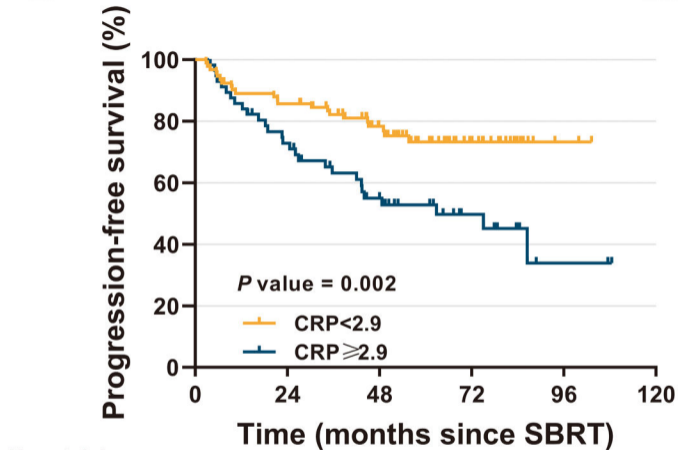

No. at risk

|           | 0  | 24 | 48 | 72 | 96 | 120 |
|-----------|----|----|----|----|----|-----|
| CRP < 2.9 | 91 | 86 | 59 | 20 | 3  | 1   |
| CRP ≥ 2.9 | 57 | 41 | 25 | 12 | 3  | 1   |

No. at risk

|           | 0  | 24 | 48 | 72 | 96 | 120 |
|-----------|----|----|----|----|----|-----|
| CRP < 2.9 | 91 | 78 | 52 | 20 | 3  | 1   |
| CRP ≥ 2.9 | 57 | 40 | 26 | 12 | 3  | 1   |

Supplement: Supplementary file 1 [file diagnostics-13-00313-s001.zip › diagnostics-2114750-supplementary/Supplement Figure S3.pdf]

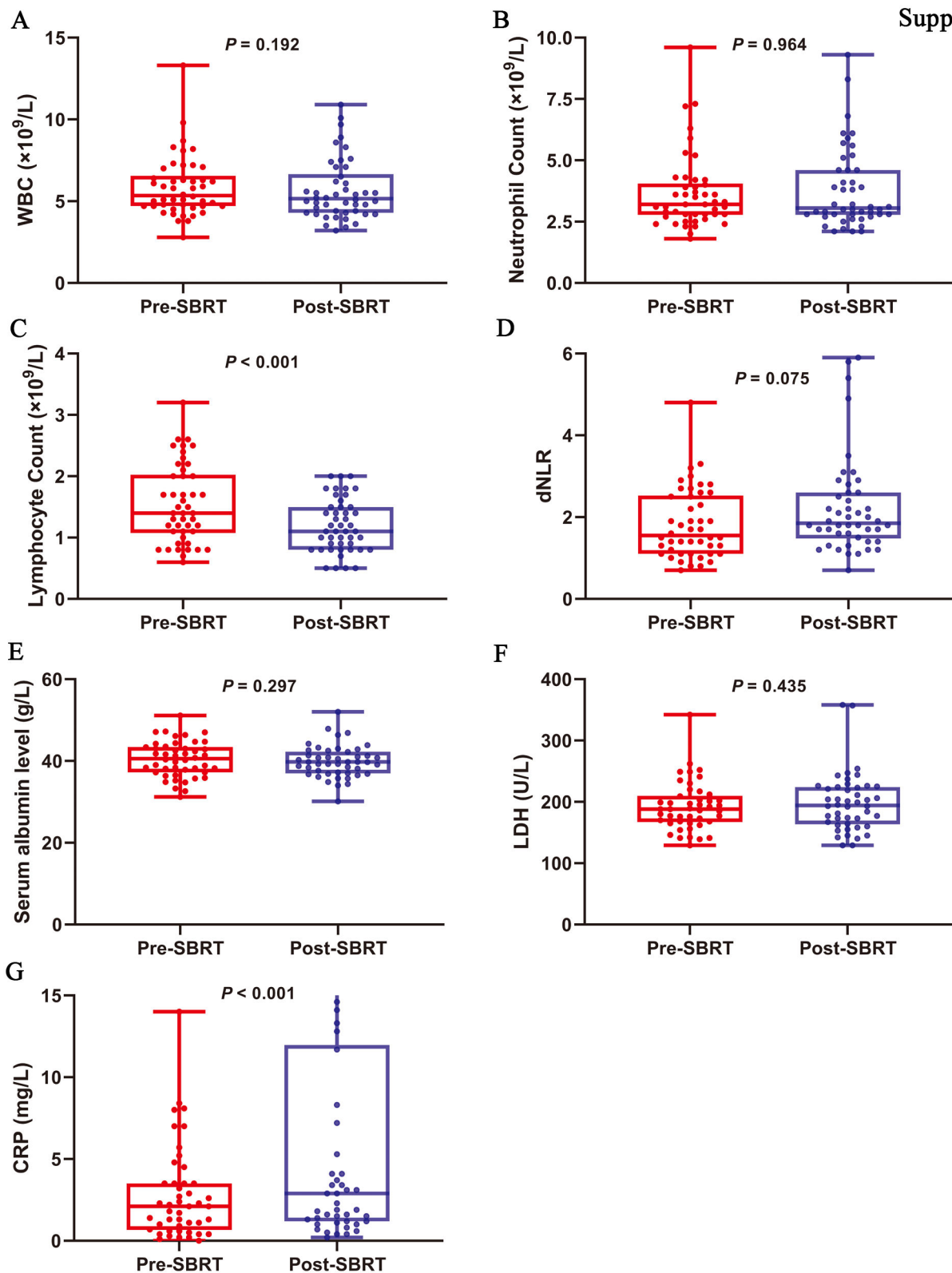

Supplement: Supplementary file 1 [file diagnostics-13-00313-s001.zip › diagnostics-2114750-supplementary/Supplement Figure S4.pdf]

A

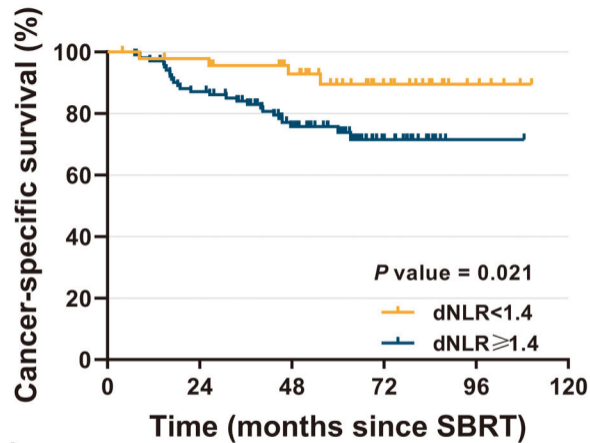

| No. at risk     |     |    |    |    |   |   |
|-----------------|-----|----|----|----|---|---|
| dNLR < 1.4      | 46  | 44 | 35 | 19 | 6 | 1 |
| dNLR $\geq$ 1.4 | 102 | 88 | 55 | 17 | 2 | 1 |

B

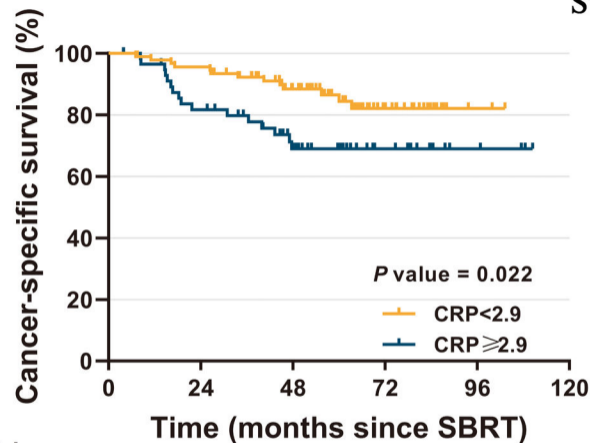

| No. at risk    |    |    |    |    |   |   |
|----------------|----|----|----|----|---|---|
| CRP < 2.9      | 91 | 87 | 60 | 21 | 3 | 1 |
| CRP $\geq$ 2.9 | 57 | 45 | 30 | 15 | 5 | 1 |

Supplement: Supplementary file 1 [file diagnostics-13-00313-s001.zip › diagnostics-2114750-supplementary/Supplement Figure S5.pdf]

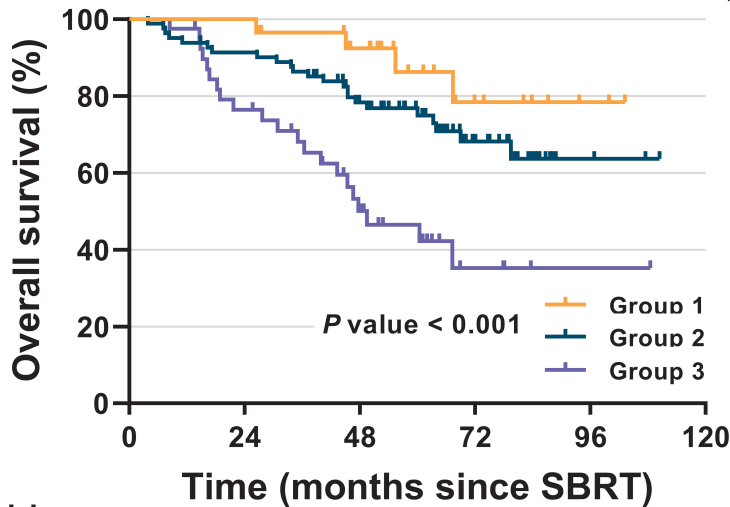

No. at risk

|         |    |    |    |    |   |   |
|---------|----|----|----|----|---|---|
| Group 1 | 28 | 28 | 21 | 9  | 3 | 1 |
| Group 2 | 81 | 74 | 54 | 23 | 4 | 1 |
| Group 3 | 39 | 30 | 16 | 5  | 2 | 1 |

Supplement: Supplementary file 1 [file diagnostics-13-00313-s001.zip › diagnostics-2114750-supplementary/Supplement Figure S6.pdf]
